# The effectiveness of interventions targeting the stigma of mental illness at the workplace: a systematic review

**DOI:** 10.1186/s12888-015-0706-4

**Published:** 2016-01-06

**Authors:** Sabine E. Hanisch, Conal D. Twomey, Andrew C. H. Szeto, Ulrich W. Birner, Dennis Nowak, Carla Sabariego

**Affiliations:** 1 Institute for Public Health and Health Services Research, Department of Medical Informatics, Biometry and Epidemiology (IBE), Ludwig-Maximilian University, Marchioninistr. 17, 81377 Munich, Germany; 2 School of Psychology, University of Southampton, SO17 1BJ Southampton, UK; 3 Department of Psychology, University of Calgary, 2500 University Dr. NW, Calgary, AB T2N 1N4 Canada; 4 Siemens AG, Human Resources, Department of Psychosocial Health and Well-being, Otto-Hahn-Ring 6, 81739 Munich, Germany; 5 Institute and Outpatient Clinic for Occupational, Social and Environmental Medicine, WHO Collaborating Centre for Occupational Health, Clinic of the Ludwig-Maximilian University, Ziemsenstr. 1, 80336 Munich, Germany

**Keywords:** Stigma, Mental health, Workplace, Intervention

## Abstract

**Background:**

The majority of people experiencing mental-health problems do not seek help, and the stigma of mental illness is considered a major barrier to seeking appropriate treatment. More targeted interventions (e.g. at the workplace) seem to be a promising and necessary supplement to public campaigns, but little is known about their effectiveness. The aim of this systematic review is to provide an overview of the evidence on the effectiveness of interventions targeting the stigma of mental illness at the workplace.

**Methods:**

Sixteen studies were included after the literature review. The effectiveness of anti-stigma interventions at the workplace was assessed by examining changes in: (1) knowledge of mental disorders and their treatment and recognition of signs/symptoms of mental illness, (2) attitudes towards people with mental-health problems, and (3) supportive behavior.

**Results:**

The results indicate that anti-stigma interventions at the workplace can lead to improved employee knowledge and supportive behavior towards people with mental-health problems. The effects of interventions on employees’ attitudes were mixed, but generally positive. The quality of evidence varied across studies.

**Conclusions:**

This highlights the need for more rigorous, higher-quality evaluations conducted with more diverse samples of the working population. Future research should explore to what extent changes in employees’ knowledge, attitudes, and supportive behavior lead to affected individuals seeking help earlier. Such investigations are likely to inform important stakeholders about the potential benefits of current workplace anti-stigma interventions and provide guidance for the development and implementation of effective future interventions.

**Electronic supplementary material:**

The online version of this article (doi:10.1186/s12888-015-0706-4) contains supplementary material, which is available to authorized users.

## Background

Despite the availability of effective mental-health treatment, the majority of people experiencing mental-health problems do not seek help. Globally, the number of people experiencing mental disorders who do not receive any healthcare treatment is estimated to reach up to 70 % [1]. This figure is alarming, given the high prevalence of mental-health problems among the general population (one in four at some point during their lifetime) [2]. Several factors have been identified that contribute to the treatment gap between true and treated prevalence: (1) lack of knowledge of the symptoms of mental illness and how to access treatment, (2) prejudicial attitudes, and (3) anticipated or real acts of discrimination against people who have mental-health problems [3–5]. These factors combined have been defined as ‘stigma’ [1], which has far-reaching consequences for those affected.

The level of accurate knowledge about mental illnesses among the general public has been reported to be rather low [6]. For example, in a population survey in England, 63 % estimated that less than 10 % of the population would be likely to experience a mental-health problem at some time in their lives [7]. However, improved knowledge of mental-health problems was shown to have a crucial effect on the ability to recognize signs of mental illness, as well as on supporting help-seeking and accepting treatment [8].

Negative attitudes or prejudice refer to negative thoughts and emotions, such as anxiety or disgust, a majority group holds against a minority group [1]. This may include public beliefs concerning mental illness, which often revolve around dangerousness and incompetence, expectations of poor prognosis, and a desire for social distance [9].

Discrimination forms the behavioral dimension of stigma and refers to any acts to the disadvantage of people who are stigmatized [10]. For example, one study reported that 47 % of the general public would not be willing to work closely with people diagnosed with depression, and 30 % would be unwilling to socialize with them [11].

Public stigma as described above can induce ‘self-stigma’ (internalization of stigmatizing attitudes), which results in diminished self-esteem and self-efficacy in people with mental-health problems [12]. The adverse effects of stigma influence various aspects of life and add an additional burden on those already dealing with a mental illness [13]. Perhaps most devastating is the impact of stigma as a major barrier to accessing mental-health treatment [14, 15].

Although the stigma of mental illness has been extensively researched among the general population, little is known about its prevalence and consequences at the workplace. Some studies suggest that the stigma of mental illness may also be an important contributing factor to the underutilization of healthcare services at work [16]. Kim et al. [17] found that soldiers failed to get treatment for Posttraumatic Stress Disorder (PTSD) because they were concerned about being stigmatized by others for having mental-health problems. Similar results have been obtained in studies on white-collar workers and the utilization of an Employee Assistance Program (EAP). Walton [18] found that employees were worried that their managers would have a negative opinion of them if they were aware of their use of mental-health services. Moreover, employees were reluctant to use counseling services at work if they believed it would negatively affect their career opportunities [19]. This clearly illustrates that the stigma of mental illness has a negative impact on the utilization of healthcare services at work and results in employees waiting until their symptoms severely interfere with their daily functioning instead of seeking support early [20]. Stigma not only poses a barrier to mental-health treatment after the onset of illness, but also interferes with prevention efforts during early stages of the illness [21].

The debilitating impact of mental illness at work is widely recognized, and resulting total work loss due to absenteeism, presenteeism, and turnover is estimated to cost organizations in the UK £26 billion a year [22]. Given the high prevalence of mental-health problems in the general and working population, the workplace is increasingly being recognized as an important target of mental-health promotion, prevention, and interventions [23]. Those efforts may remain ineffective if stigma is not removed and a supportive work environment is not created. Therefore, effective strategies to reduce the stigma of mental illness and to increase help-seeking behavior at the workplace are needed. Unfortunately, research on their effectiveness is scarce and presents inconclusive evidence in this field [23, 24].

Although six systematic reviews investigating anti-stigma reduction programs have been conducted, none of them focused specifically on workplace interventions [25–30]. Two non-systematic reviews of current workplace anti-stigma programs were published, but they were mainly conceptual in nature rather than evaluating the effectiveness of the interventions [23, 24]. Insight in improve existing efforts in the development of targeted workplace anti-stigma interventions is sorely lacking.

Such an investigation could inform important stakeholders about the effectiveness of current workplace anti-stigma interventions and their potential benefits in terms of an inferred impact on utilization rates of healthcare services (e.g. workplace counselling/EAP etc.) and on employee mental health. Such investigations could strengthen the incentive for organizations to invest in stigma-reduction efforts while providing guidance for the development and implementation of effective future interventions.

Therefore, based on the conceptual framework of stigma by Thornicroft [1] as described earlier, this review aims to provide a first systematic review on the effectiveness of workplace anti-stigma interventions by examining changes in: (1) knowledge of mental disorders and their treatment and recognition of signs/symptoms of mental illness, (2) attitudes towards people with mental-health problems, and (3) supportive behavior among colleagues (e.g. reduced discriminatory or increased affirmative behavior, help seeking, etc.). We chose to adhere to this conceptualization because, in contrast to the majority of evaluation studies, we wanted to place particular emphasis on measuring behavioral outcomes of stigma-reduction programs and help-seeking [3, 28, 31].

## Methods

A systematic literature review on the effectiveness of workplace anti-stigma interventions was carried out after methods of the analysis and inclusion criteria had been specified in a protocol.

### Eligibility criteria

For detailed information on eligibility criteria, please refer to Additional file 1 in the supplementary material.

#### Study designs of interest

Randomized controlled trials and quasi-experimental studies were included, while longitudinal studies, cohort studies, primary prevention studies, phase-I and II studies, ecologic studies, case reports, case series, cross-sectional studies, and qualitative and economic evaluations were excluded from the analysis. This is because, in contrast to previous descriptive reviews on anti-stigma interventions, this review aimed to focus exclusively on evaluating the effectiveness of workplace anti-stigma programs and, thus, only included experimental studies which provided quantitative evidence.

#### Study participants

Participants aged 18–65 in the working population were considered. Studies that targeted mental healthcare providers were excluded from this review because this occupational group already has extensive knowledge of and contact with people with mental-health problems. Preliminary evidence suggests that this group might be fundamentally different in their responses to anti-stigma interventions than people working outside of healthcare [32]. Studies targeting self-stigma in clinical patients were also excluded.

#### Types of interventions

All types of interventions targeting stigma against mental illness at the workplace were considered for the current review. Studies were included if they met the following criteria: (1) included an intervention that targeted at least one dimension of stigma as an outcome (any variables related to either knowledge and/or attitude and/or behavior were considered), (2) included an evaluation of the intervention, and (3) the evaluation was quantitative. This meant that programs which targeted any dimension of stigma were included, even though they couldn’t necessarily be considered anti-stigma programs per se.

Studies were excluded if they met the following criteria: (1) self-stigma in clinical patients was targeted, (2) did not include an evaluation of the intervention, or (3) presented only qualitative evaluation data.

### Information sources

Medline and PsycINFO were searched for peer-reviewed articles related to workplace anti-stigma interventions carried out between 2004 and 2014. This time span was considered exhaustive enough to include the most recent efforts, as well as those started ten years ago. Only papers in the English, German, Spanish, or Portuguese languages could be read and were selected. References in relevant articles were also screened for publications that might be acceptable for inclusion. An additional Google Scholar search was made to identify relevant grey literature, which is either unpublished or not published in peer-reviewed journals. Experts at the Mental Health Commission of Canada were also consulted for the potential inclusion of unpublished articles.

The last search was run on July 1, 2014.

### Search strategy (see Additional file 1)

The search strategy was reviewed independently by subject experts/librarians at the University of Calgary (for full database search strategies, please check the *appendix*). The following terms were used to search all trial registers and databases: stigma-related terms AND mental health-related terms AND workplace-related terms AND program evaluation-related terms. Limitations were applied with regards to restrictions in type of study design and type of participants as described above, as well as to studies on stigma related to physical health conditions or interventions aiming to reduce drug use (e.g. smoking cessation) unless they provided a quantitative measure on stigma related to drug use and didn’t target healthcare providers.

Stigma-related terms: stigma*, labeling, prejudice, social acceptance or social approval, social discrimination, social perception, stereotyped attitudes, shame, discrimination or disability discrimination, judgment, fairness, health services accessibility, treatment barriers.

Mental health-related terms: mental disorders, psychiatric patients, psychiatric symptoms, recovery disorders, relapse disorders, work-related illnesses, mental health, well-being.

Workplace-related terms: occupations, employment history, occupational adjustment, occupational tenure, personnel, professional personnel, working women, employment status, employability, reemployment, supported employment, occupational health, industrial and organizational psychology, working conditions, unemployment, personnel termination, downsizing, workplace*, quality of work life, occupational stress, organizational climate.

Program evaluation-related terms: mental illness (attitudes toward), mental health program evaluation or mental health programs, community mental health training or mental health inservice training or inservice training or professional development, program development, program evaluation, health promotion, health education or health knowledge or health literacy or social marketing or client education, structured clinical interview or interviews or psychodiagnostic interview or interviewers or interviewing or qualitative research or questioning or narratives or life review or narrative therapy or storytelling or health attitudes or attitudes or disabled (attitudes toward) or employee attitudes or employer attitudes or health personnel attitudes, or occupational attitudes or public opinion or work (attitude toward) or attitude measurement or attitude measures, campaign or initiative or aware or program or train or intervene or workshop or seminar or curriculum or booster session or strategy or implement or course or symposium or coach or mentor or blitz or policy or policies or guideline or recommendation or standard, questionnaires or mail surveys or surveys or telephone surveys.

### Study selection

An eligibility assessment of abstracts and full-text papers was performed in a standardized manner by the lead author (SH), and 20 % of total citations were double checked independently by a second reviewer (CT). Disagreements between reviewers were followed up by double checks and resolved by discussion.

### Data extraction

Data on study design, sample characteristics, and findings were extracted by two reviewers (SH, CT) independently (CT double extracted 31 % of total full-text inclusions). The following information was extracted from each included study: (1) objectives, (2) general information (study design, country of origin, number and duration of follow-ups), (3) study population (age, sample size, percentage of female participants, target population), (4) workplace (workplace name, workplace sector, workplace type, job, (5) type of intervention (duration, frequency, target in terms of primary- and secondary- outcome measures, and whether the intervention addressed general mental health or a specific mental illness), and (6) intervention effectiveness (in terms of a change in outcome measures with effect sizes where reported). No further variables were added to those already pre-specified in the protocol after the review had begun.

### Study quality

For all included studies (including grey literature), methodological quality was assessed using a checklist for randomized controlled trials and quasi-experiments [33]. This checklist involved an assessment of four kinds of systematic errors (detection, selection, attrition, and information bias) among a rating scale of low, moderate, or high risk. Two authors (SH, CT) independently rated all studies according to those criteria and resolved discrepancies through discussion. If no agreement could be reached, a third author was consulted.

### Data analyses

A narrative synthesis following the guideline proposed by Popay et al. [34] was undertaken since a meta-analysis of results was not possible due to substantial differences in methodology and outcome data across studies. This involved addressing four main elements of narrative synthesis: a) developing a theory of how the intervention works, why, and for whom, b) developing a preliminary synthesis of findings of included studies, c) exploring relationships within and between studies, and d) assessing the robustness of the synthesis. Extracted information was summarized using the tabular form of the Cochrane review’s ‘Characteristics of Included Studies’ table (participants, interventions, outcomes, notes) with the inclusion of additional information (country of origin, duration of the intervention, target, assessment time points, control group, study design, and the context in which the intervention was delivered).

## Results

### Study selection

**Fig. 1:**
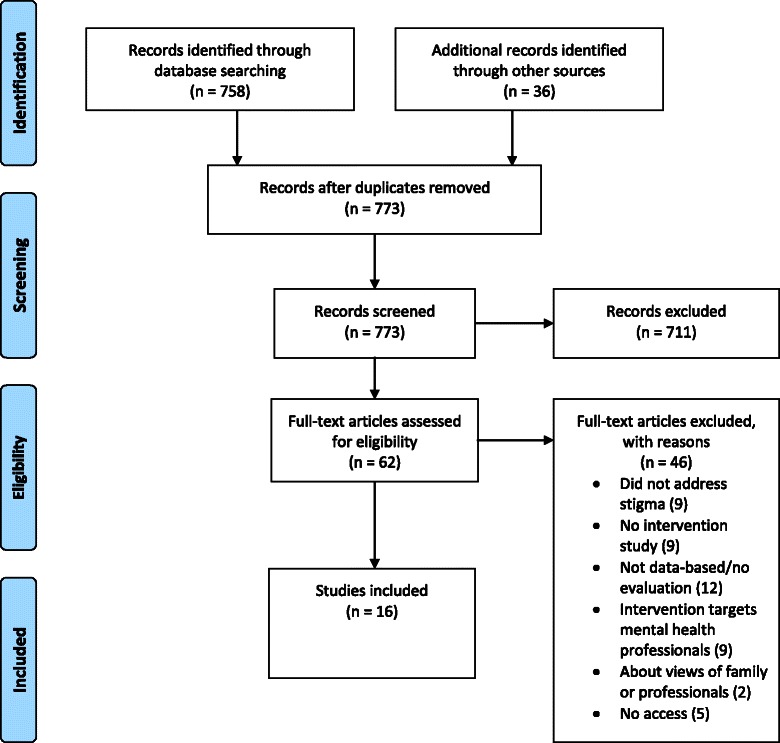
Preferred Reporting Items for Systematic Reviews and Meta-Analyses (PRISMA) flow diagram. Study selection process

The study selection process was carried out according to the PRISMA guidelines on reporting items for systematic reviews [35]. Appropriate studies were identified in Medline and PsycINFO (yielded 758 citations), while 36 additional citations were identified searching Google Scholar, consulting experts of the Mental Health Commission of Canada, and by checking the references of relevant papers. Seven hundred seventy-three studies remained after duplicates were removed. Seven hundred eleven were excluded since they clearly did not meet the criteria after abstract review. After reviewing the full text of the remaining 62 citations, 46 studies were excluded for specific reasons which are listed in the flow chart (see Fig. 1). Sixteen studies were eventually included in the review.

### Study characteristics

For detailed information on study characteristics, please see Additional file 2 in the supplementary material.

#### Study designs

Of the 16 included studies, five were RCTs, and 11 were quasi-experimental studies. Seven studies included a control group. All studies were published in English.

### Settings and populations

The included studies involved 3854 participants. The majority of studies targeted the public sector (12), only two the private sector, and no information on the type of workplace was given for another two. Regarding study populations, most studies examined interventions for managers or supervisors, as well as first responders, such as police officers. Two studies [36, 37] examined interventions in employees routinely working with people with mental-health problems (e.g. housing agencies). Six studies were conducted in Europe, five in the US or Canada, four in Australia, and one in Asia.

### Interventions

Eight studies assessed the impact of Mental Health First Aid (MHFA) training or a modified version of the program on one or more dimensions of stigma. While MHFA is primarily seen to be a mental-health literacy program, they do measure stigma and were therefore included [24]. The remaining eight studies included heterogeneous interventions, such as role play, online training, psychoeducation, workshops, Trauma Risk Management (TRiM), and Crisis Intervention Training (CIT) in first responders. Half of the studies targeted all three dimensions of stigma (i.e. knowledge, attitudes, behavior), while the other half specifically targeted attitudes or behavior. The duration of the interventions varied between a minimum of 1 hour up to a maximum of 2 days.

### Outcomes

#### Primary

In all studies the primary outcome was a change in at least one dimension of stigma, namely knowledge and/or attitude and/or behavior. While studies differed with regards to the operationalization of variables for knowledge, attitude, and behavior outcomes, data collection and instruments used to assess change over time were fairly similar across studies.

#### Secondary

Secondary outcomes included change in participants’ overall mental health [38, 39]. One study examined readiness to provide actual help to people with mental disorders as the primary outcome while analyzing knowledge, attitudes, and self-confidence in helping a person with a mental disorder as secondary outcomes [40]. One study assessed the cost-effectiveness of the intervention [41].

### Study quality

In general, all studies included were considered at high risk for detection bias, as at least one dimension of stigma was measured by self-reports (which is, however, fairly standard and about the only feasible way to measure attitudes). With regards to selection, attrition, and information bias, the majority of studies was at high risk of bias due to selective reporting, lack of allocation concealment, lack of participant blinding, and incomplete outcome data. While five studies received an overall rating of low risk for bias, no study had only low risk of bias ratings for the type of bias described above.

### Effectiveness of anti-stigma interventions

See Table 1 for a summary of results of the included studies with regards to intervention effectiveness (for reported effect sizes, please refer to Additional file 3).

**Table 1:** Overview of results of the included studies with regard to intervention effectiveness

Author (Year)	Intervention type	Knowledge*,**	Attitudes*,**	Behavior*,**	Success rate^a^
Svensson & Hansson (2014) [40]	Mental Health First Aid (Adult)	T C	T	T C	2/3 (67 %)
Krameddine et al. (2013) [41]	Role plays	C		T C	1/1 (100 %)
Hossain et al. (2009) [42]	Mental Health First Aid (Adult)	T C	T C	T C	3/3 (100 %)
Massey (2010) [43]	Mental Health First Aid (Adult)	T C	T	T C	2/3 (67 %)
Kitchener & Jorm (2004) [38]	Mental Health First Aid (Adult)	T	T C	T C	2/3 (67 %)
Luong et al. (2013) [51]	Online Training, Group discussions		T		0/1 (0 %)
Gould et al. (2007) [39]	Trauma Risk Management		T C		1/1 (100 %)
Stuart et al. (2013) [50]	Online Training		T C	T C	2/2 (100 %)
Knifton & Quinn (2009) [36]	Anti-stigma workshop	T C	T C	T C	3/3 (100 %)
Nishiuchi et al. (2007) [47]	Psychoeducation	T C	T	T	1/3 (33 %)
Compton et al. (2006) [44]	Crisis Intervention Training	T C	T C		2/2 (100 %)
Moffitt et al. (2014) [48]	Training course or Mental Health First Aid vs. leaflet session	T C	T C	C	2/2 (100 %)
Quinn et al. (2011) [37]	Anti-stigma workshop		T C		1/1 (100 %)
Jorm et al. (2010) [49]	Mental Health First Aid (Youth)	T C	T C	T C	3/3 (100 %)
Pierce et al. (2010) [45]	Mental Health First Aid (Youth)	T C	T	T C	2/3 (67 %)
Brandling & McKenna (2010) [46]	Mental Health First Aid (Youth)	T C		T C	2/2 (100 %)

^a^Success rate = targets successfully changed/total targets

*T = outcome targeted by intervention

**C = change occurred, intervention success

#### Knowledge

Eleven studies targeted ‘knowledge’, including a) the identification of mental-health problems and b) knowledge about effective treatments.

Ten anti-stigma interventions were shown to be effective in increasing mental-health knowledge with one exception. In this study, MHFA training did not result in improved mental-health literacy in the intervention as opposed to the control group [38]. However, since recognition of a mental disorder in a vignette task was already fairly high in the pre-test, this left limited room for improvement post intervention. Six studies with high risk of bias had a positive impact on mental-health literacy [36, 42–46]. Nevertheless, the impact of their evidence is weaker given the absence of a control group, the lack of randomization procedures, and a high risk of selection bias (e.g. participation in the intervention was voluntary). These findings are supported by other studies of moderate-to-high quality, which confirms a significant positive effect of workplace anti-stigma interventions on employees’ mental-health knowledge [40, 47–49].

#### Attitudes

Fourteen studies measured stigmatizing attitudes or openness towards people or coworkers with mental illness, often using social-distance scales. One study examined specific attitudes related to perceived dangerousness, unpredictability, and recovery of mentally-ill individuals [36]. Another study differentiated between first- and third-person viewpoints with regards to stigma [37].

Although the effectiveness of interventions on changing attitudes was mixed, nine studies did report improvements in participants’ stigmatizing attitudes. Next to the MHFA training, the other types of anti-stigma interventions, such as TRiM, CIT, online training, and workshops, were effective in reducing stigmatizing attitudes towards people with mental-health problems. Of the six studies with low-to-moderate risk for bias, four reported a significant positive effect on participants’ attitudes [38, 39, 48, 49], while two did not note any significant changes [40, 47]. However, while Svensson, Hansson [40] found no overall significant change in attitudes, their analyses of specific items on their stigma scales did reveal positive improvements (e.g. items related to personal stigma, as well as becoming a neighbor of a person with depression). With regards to more specific attitudinal changes, Knifton et al. [36] found particular improvement in relation to unpredictability and recovery, but not for dangerousness.

#### Behavior

Eleven studies targeted ‘behavior’. Behavior was operationalized in a heterogeneous manner across studies, including both true behavioral measures and proxies. In general, behavioral change was related to increased affirmative behavior, as well as to reductions in discriminatory behavior.

All types of anti-stigma interventions in 11 studies (three rated as of high quality) consistently had a significant positive impact on employees’ supportive behavior [36, 38, 40–43, 45, 46, 49, 50] with the exception of one study [47], which reported a marginally significant effect. More specifically, this involved, for example, perceived confidence and self-efficacy in identifying and dealing with a person with a mental illness, as well as the likelihood of advising people to seek professional help and readiness to provide help in mental-health situations. One study involving police officers examined directly measured behaviors, such as the use of force [41].

In one study, role play was used to achieve behavioral change. Although not intended, the intervention also had a positive effect of mental-health knowledge [41]. Similarly, Moffitt et al. [48] observed a change in behavior achieved by an intervention that targeted knowledge and attitude only.

#### Secondary outcomes

Two studies of moderate to high quality examined participant mental health as a secondary outcome and reported a positive impact of the anti-stigma intervention [38, 39]. The study including a cost-effectiveness analysis found its anti-stigma intervention (i.e. role play) to be cost-effective [41].

### Sustainability of change

Eleven studies did not include any follow-up measurements beyond the initial two time points (pre-post). This limits the conclusions that can be drawn relating to the effectiveness of anti-stigma interventions over the long term. Five studies conducted a post-intervention follow-up of up to 2 years [39, 40, 47, 49, 51]. All these studies report that the changes achieved in either people’s knowledge and/or attitudes and/or behavior post-intervention were, in part, sustained over time. For example, Svensson, Hansson [40] found a significant improvement in knowledge and confidence to provide help, but not in attitudes, and this pattern remained unchanged at a 2-year follow-up.

## Discussion

To our knowledge, this is the first systematic review to examine the effectiveness of interventions targeting stigma towards mental illness at the workplace. The majority of the included studies were published since 2010, reflecting a growing interest in evaluations of stigma-reduction programs at the workplace. Our review illustrates that workplace anti-stigma interventions may be effective in changing employees’ knowledge, attitudes, and behavior towards people with mental-health problems. However, due to methodological shortcomings in the majority of the included studies, the lack of follow-ups beyond post-intervention assessments, as well as heterogeneity in terms of intervention content, duration, and outcome measures, the evidence for the effectiveness of workplace anti-stigma interventions is inconclusive and must be interpreted with caution.

While prior systematic reviews of general population interventions corroborate our findings of poor evaluation study design, they also found stigma-reduction efforts to be effective in changing people’s knowledge, attitudes, and behaviors towards people with mental-health problems [25–29]. The development and implementation of effective anti-stigma programs specifically designed for the workplace is, however, of high importance. First, while public efforts have returned mixed results, the development of tailored strategies targeting the workplace might prove a more promising route to stigma change, as awareness of public campaigns has often been found to be quite low [24, 52]. Thus, while public anti-stigma efforts target a greater part of the population, more people might be reached effectively via more targeted interventions (e.g. at work). Second, participation in anti-stigma programs, for example in the scope of personnel development, could be made mandatory in an organizational setting, whereas public stigma campaigns require people to participate voluntarily. Third, by nature, exposure to mass-media approaches to stigma change can be short in time, whereas workplace interventions can be more intensive in terms of length and information.

Our review shows that workplace anti-stigma interventions can be particularly effective in changing employees’ knowledge of mental disorders, as well as helping behavior, while results related to attitudinal change were mixed, but positive overall. In two studies [41, 48], a spillover effect was identified, meaning that a change in one outcome measure (e.g. behavior) occurred even though the intervention exclusively targeted other outcomes (e.g. knowledge or attitudes). This implies that the three dimensions of stigma (knowledge, attitude, and behavior) might be interrelated, as has been suggested before [53]. The theory of health education [54] postulates that attitude mediates the relationship between knowledge and behavior. In contrast, the current review showed that attitudinal change is not required to achieve behavioral change. In line with prior research [47, 55], three studies found that knowledge might directly trigger a behavior under certain conditions, even without any attitudinal change [40, 43, 45]. However, further research into how anti-stigma interventions change or affect each of the three dimensions of stigma is required to fully understand the stigmatization process.

The debilitating impact of mental illness at work is widely recognized, and organizations are increasingly investing in workplace mental-health interventions. However, emerging evidence indicates that stigma towards mental illness, in part, contributes to the underutilization of costly mental-health services (e.g. EAP, workplace counseling) that are already offered by organizations [16, 18]. It is, therefore, important to address and remove stigma as a barrier to increase the effectiveness and ‘value-for-money’ of these interventions.

This review addresses the research gap regarding the behavioral dimension of stigma as an outcome and, more importantly, highlights that workplace anti-stigma interventions have the potential to change employee behavior [3]. In contrast, anti-stigma campaigns targeting the general public have often failed to change behavior [56]. Perhaps in an organizational context as compared to the public context, behavioral change (e.g. in supportive or help-seeking behavior) could be achieved more readily by giving clear calls for action in specific situations at work. This has important practical implications for organizations and employers alike, as behavioral change is considered the ultimate goal of efforts to reduce stigma and is likely to result in a more supportive work environment, which, in turn, is a necessary prerequisite for the success of any mental-health intervention (e.g. workplace counseling, EAP) [53, 57].

In light of the impact of stigma on seeking help and accounting for the fact that a large proportion of people experiencing mental-health problems do not seek help, it is essential to measure the impact of anti-stigma interventions on help-seeking behavior [58]. Despite the heterogeneity in the operationalization of behavior, however, none of the included studies examined help-seeking behavior as an outcome, focusing instead on potential intervention effects on participants’ supportive behavior towards afflicted individuals. Future evaluations of workplace anti-stigma interventions should place stronger emphasis on assessing a potential impact on employees’ help-seeking behavior (e.g. health-service utilization), as well as on their mental health (e.g. sick leave, presenteeism). This would help assess the cost-effectiveness of workplace anti-stigma interventions and strengthen the economic incentive for organizations to invest in stigma-reduction efforts.

The current review found some evidence indicating the positive impact of anti-stigma interventions on participants’ general mental health [36, 37]. Improved knowledge of signs of mental illness and treatment options may lead employees to seek help earlier. This is supported by findings of a prior meta-analysis, which found that MHFA training helped improve participant mental health by improving self-recognition, increasing insight into one’s own and others’ mental well-being, and by increasing coping skills [30]. Workplace anti-stigma interventions might not only create a more supportive work environment by reducing stigmatizing attitudes and discrimination, but also lead to improved knowledge and awareness of mental illness and to improved employee mental health via increased and potentially earlier help-seeking. So far, economic evaluations of anti-stigma interventions are generally lacking; however, preliminary evidence indicates a potential return on investment for employers [59].

While the evaluated anti-stigma interventions themselves seem to be scientifically sound in terms of their theoretical background and content, the evaluation methods used need to be improved substantially. A prominent finding of this review was the large number of studies with methodological shortcomings, high risk of bias, no control groups, and small sample sizes. Studies frequently also reported high levels of dropouts and varied in terms of program completion. A potential reason for this might be the challenge of evaluating interventions in a scientifically sound manner in companies which might be unwilling to engage in such research or pose restrictions due to data-protection rights.

The current review further highlights a misfit between what some intervention studies claimed to target and what they actually assessed in terms of outcomes [41, 48]. If studies fail to assess the impact on outcomes they claim to target in their intervention, important evaluation data gets lost. Studies targeting and assessing a change in only one dimension of stigma (e.g. attitude) might fail to detect a spillover effect on other dimensions of stigma (e.g. knowledge or behavior).

Previous research has questioned the retention of intervention effects over time, especially with regards to attitudinal and behavioral change [28, 29]. The majority of studies in this review did not conduct a follow-up assessment of intervention effects. However, where reported, improvements in knowledge, attitudes, and behavior were maintained over time [39, 40, 47, 49, 51]. Future research needs to place greater emphasis on conducting follow-up evaluations that go beyond pre-post measurements.

### Limitations

Although this review generated important findings, there are several limitations that should be mentioned. First, only three electronic databases were used to gather articles for this review, and a search in languages other than English, German, Portuguese, and Spanish was not undertaken. Despite the lack of breadth, the searches were supplemented by searching Google Scholar, checking references, and communication with experts, which yielded 14 further studies, three of which were unpublished. The possibility of publication bias needs to be considered, as there may have been relevant studies that did not produce positive results and, consequently, were not published.

A second limitation of the current review involves generalizability of the current findings. The majority of participants in the reviewed studies were well-educated employees, such as managers. This limits the generalizability of the findings to other occupations or sectors that employ less-educated workers (e.g. service industries). While it makes sense to address managers due to their supervisory role and their importance in recognizing and dealing with signs of mental illness in subordinates, it may be just as important to target less-educated workers because there is some evidence indicating that less-educated compared to more-educated people are more likely to hold stigmatizing attitudes towards people with mental illnesses [60]. It is also important to note that all of the studies included in this review were carried out in high-income countries and, therefore, the findings may not apply to low- and middle-income countries, where stigma towards mental illness might be particularly strong or prevailing.

This review provides a narrative synthesis of the evidence of anti-stigma intervention effectiveness rather than a meta-analysis of results, which limits the strength of the conclusions that can be drawn. Given the heterogeneity of the methodology and outcome data across studies, it was not possible to conduct a meta-analysis at this time.

### Implications for future research

It was beyond the scope of the current review to identify which types or components of anti-stigma interventions are particularly effective in improving employees’ knowledge, attitudes, and behavior. Future research should compare and contrast different types of anti-stigma interventions to determine the optimal program content and duration for the workplace context. Although a positive impact was found in all types of anti-stigma interventions studied, it is crucial to emphasize a stronger evaluation methodology as much as improving anti-stigma content.

Future research in this field should engage in more standardized, high-quality evaluations which measure all dimensions of stigma towards mental illness to better understand the potential impact of anti-stigma interventions at the workplace. This would allow researchers to compare quantitative measures of stigma across studies more easily and to conduct a meta-analysis which would help build a stronger evidence base for the effectiveness of workplace anti-stigma interventions.

To increase the generalizability of the current findings, anti-stigma interventions with larger, more diverse samples in terms of gender, race, socioeconomic status, education/hierarchy, geographic location, and type of workplace should be tested.

## Conclusions

This review systematically examined the effectiveness of interventions targeting stigma towards mental illness at the workplace. There is tentative evidence that workplace anti-stigma interventions can have a positive impact on employees’ knowledge, attitudes, and supportive behavior towards people with mental illness. The quality of evidence varied across studies, highlighting the need for more rigorous, higher-quality evaluations conducted with more diverse samples of the working population.

Future research needs to explore to what extent changes in employees’ knowledge, attitudes, and supportive behavior translate into increased and earlier help-seeking by affected individuals. Such investigation is likely to inform important stakeholders, like human-resources or health-management personnel, about the beneficial impact of stigma-reduction programs on the effectiveness or acceptance of already existing mental-health interventions and, ultimately, on employee mental health.

## Additional files

Additional file 1: **Appendix.** Primary search strategy for MEDLINE. Primary search strategy for PsycINFO. Selection criteria (inclusion & exclusion). (XLSX 15 kb)
Additional file 2: Table S1. Overview of study characteristics of included studies. (XLSX 15 kb)
Additional file 3: Table S2. Effect sizes reported for included studies. (XLSX 13 kb)

## Abbreviations

PTSD: posttraumatic stress disorder
EAP: employee assistance program
RCT: randomized controlled trial
MHFA: mental health first aid
TRiM: trauma risk management
CIT: crisis intervention training
